# The Effects of Slow Breathing during Inter-Set Recovery on Power Performance in the Barbell Back Squat

**DOI:** 10.5114/jhk/185935

**Published:** 2024-05-17

**Authors:** Jeffrey D. Buxton, Holly M. Grose, Joseph D. DeLuca, Troy P. Donofrio, Vincent R. LePre, Clayton W. Parrish, Hayden D. Gerhart, Philip J. Prins

**Affiliations:** 1 Department of Exercise Science/Grove City College, Grove City, PA, USA.

**Keywords:** velocity, resistance training, parasympathetic nervous system

## Abstract

Slow breathing (SB) reduces sympathetic nervous system activity, the heart rate (HR), and blood pressure (BP) and increases parasympathetic nervous system activity, HR variability, and oxygen saturation which may lead to quicker recovery between bouts of exercise. Therefore, the purpose of this study was to examine whether a SB technique using the 4-7-8 method between sets of barbell back squats (SQs) would attenuate drops in power and bar velocity. In a randomized, crossover design, 18 healthy resistance-trained college-aged males (age: 20.7 ± 1.4 years, body height: 178.6 ± 6.4 cm, body mass: 82.2 ± 15.0 kg, 4.5 ± 2.4 years of experience) performed 5 sets of 3 repetitions of SQs with normal breathing (CON) or SB during the 3-min recovery between sets. Peak and average power and bar velocity were assessed using a linear positioning transducer. HR recovery (RHR), systolic BP recovery (RBP), the rating of perceived exertion (RPE) and the rating of perceived recovery score (RS) were assessed after each set. There were no significant differences between conditions for peak and average power and bar velocity, RBP, RPE, and RS (p > 0.211). SB led to a greater RHR after set 2 (SB: 51.0 ± 14.9 bpm vs. CON: 44.5 ± 11.5 bpm, p = 0.025) and 3 (SB: 48.3 ± 13.5 bpm vs. CON: 37.7 ± 11.7 bpm, p = 0.006) compared to the CON condition. SB was well tolerated, did not hinder nor improve training performance and improved RHR after the middle sets of SQs. Further investigations are warranted to examine the effects of other SB techniques and to determine SB’s effects on different training stimuli as well as its effects over an entire workout and post-workout recovery metrics.

## Introduction

Power, defined as the rate of work or product of force times velocity, is a critical characteristic of many sports (Hester et al., 2014; Kawamori and Haff, 2004) and therefore the subject of much research attempting to identify optimal training strategies (Baker and Newton, 2007; Cormie et al., 2007; Thomas et al., 2007; Weakley et al., 2019). Resistance training for power is an accepted strategy that generally involves complex movements with submaximal loads (30 to > 80% 1RM) performed at maximal velocities (e.g., the barbell bench press, the back squat and Olympic lifts) (Cormie et al., 2007; Kawamori and Haff, 2004; Thomas et al., 2007). With advances in technologies, the role of repetition velocity has received increased attention (Weakley et al., 2019) with some research suggesting greater athletic improvement when training at maximal intended velocities (González-Badillo et al., 2014; Pareja-Blanco et al., 2014). During multiple set training, power and velocity reductions within a set and from set to set are expected (Hester et al., 2014; Weakley et al., 2019). Attenuating these reductions in power or velocity from set to set during resistance training may improve power training related outcomes.

Aside from the manipulation of duration of rest between sets, several novel strategies have been evaluated as potential ergogenic aids for attenuating within-set and set-to-set performance reductions including visual feedback (Weakley et al., 2019), site specific cooling strategies (Grahn et al., 2012; Kwon et al., 2010; Wu et al., 2023) and cyclic hyperventilation between sets (Buxton et al., 2022; Sakamoto et al., 2020), each with mixed results. Palm and foot cooling and visual feedback have demonstrated an ability to attenuate training decrements compared to control conditions, however, these strategies may be cost prohibitive for certain athletes. Between-set breathing techniques therefore present affordable and easily accessible strategies.

The use of various breathing strategies during resistance exercise has been shown to positively modify internal body pressures (e.g., intra-abdominal pressure, inter-thoracic pressure) which can enhance joint stability and ultimately improve exercise performance (Blazek et al., 2019, 2020). The effectiveness of inter-set breathing techniques on exercise performance is less understood. The work of Sakamoto’s lab has identified cyclic hyperventilation between sets as a method for attenuating drops in power during repeated cycling sprints (Sakamoto et al., 2013) and increases in repetitions to failure (range: +21.3 to 55.7%) and repetition velocity (range: 6.3 to 15.3%) in the bench press and leg press exercises (Sakamoto et al., 2020) in well-trained athletes, primarily through mitigation of exercise induced acidosis. However, Buxton et al. (2022) found no influence on repetitions to failure, power, or bar velocity using a similar breathing technique in lesser-trained individuals suggesting that training experience may play a role.

Hyperventilation generally increases sympathetic nervous system (SNS) activity and work of the ventilatory muscles, which may explain the null findings in the lesser trained cohort of the previous study (Buxton et al., 2022). Slow breathing (SB) techniques (e.g., 4-7-8 breathing) tend to reduce SNS activity, the heart rate, and blood pressure and increase parasympathetic nervous system (PSNS) activity, heart rate variability (HRV), and oxygen saturation (Magnon et al., 2021; Mason et al., 2013; Russo et al., 2017) without increasing the ventilatory muscle workload and therefore being more suitable for moderately trained individuals. Recently Vierra and colleagues (2022) demonstrated the effectiveness of the 4-7-8 breathing method (4-s inhale, 7-s pause, 8-s exhale) for acutely reducing the HR and blood pressure and altering HRV domains suggestive of increased PSNS activity (i.e., reduced low frequency and very low frequency power and increased high frequency power) (Vierra et al., 2022). These effects may lead to quicker recovery between bouts of exertion than normal breathing and therefore attenuate normal performance decrements within a set and between sets. To our knowledge there is limited research on the use of PSNS stimulating SB strategies, such as the 4-7-8 method, as an inter-set recovery aid for power training. The 4-7-8 method, like most other forms of SB, is a highly accessible and affordable potential training optimization strategy that could be used by almost any population.

Therefore, the purpose of this study was to examine whether a PSNS emphasized SB technique using the 4-7-8 method between sets of barbell back squats (SQs) would attenuate drops in power and bar velocity in moderately trained college-aged men. We hypothesized that the 4-7-8 breathing method used between sets would result in reduced power and bar velocity decrements, and lower recovery heart rates (HRs), blood pressure (BP) and the rating of perceived exertion (RPE) compared to normal breathing.

## Methods

### Experimental Approach to the Problem

A randomized, counter-balanced, cross-over design was used to investigate the effects of SB using the 4-7-8 method performed between sets of SQs on various measures of power and bar velocity, the HR, BP and the RPE. Participants’ 1 repetition maximum (1RM) for the SQ was determined during a familiarization session. Following familiarization, participants completed two training sessions separated by seven days involving a standardized warm-up and 5 sets of 3 repetitions of SQs performed at 80% of their 1RM with 3-min rest intervals. One session consisted of using the 4-7-8 slow breathing (SB) technique between sets, while the other used normal breathing (CON). Bar velocity and power were assessed for each repetition using a Tendo Power Unit. The RPE was measured after each set, while blood pressure (BP) was measured manually immediately after each set and 2 min into the 3-min recovery period. The heart rate (HR) was monitored continuously throughout each session using a Polar HR chest strap monitor. Rating of perceived recovery scores (RSs) were assessed at the end of the recovery period.

### Participants

Eighteen healthy resistance-trained college-aged males (Table 1) who were currently resistance training 3–5 days per week and had performed the SQ exercise with 80–100% of their 1RM within the last year volunteered. Exclusion criteria included 1) any musculoskeletal injury preventing successful and complete participation, 2) presence of known cardiovascular or respiratory disease, 3) currently competing in weightlifting competitions, 4) use of performance enhancing supplements (e.g., creatine), and 5) current participation in varsity athletics. Participant demographics are shown in Table 1. All participants were informed of the risks and benefits of participation prior to providing their written informed consent. Participants were instructed to refrain from vigorous physical activity, caffeine, and alcohol consumption in the 24 h prior to testing, and all food 3 h prior to testing. Additionally, participants were asked to maintain their normal training and nutritional habits throughout the study period. All procedures were approved by the Institutional Review Board of the Grove City College (protocol code: #118-2022; approval date: 21 December 2022).

**Table 1:** Participants’ demographics (n = 18).

Variables	Value (Mean ± SD)
Age (years)	20.7 ± 1.4
Body Height (cm)	178.6 ± 6.5
Body Mass (kg)	82.2 ± 15.0
Body Fat (%)	15.0 ± 6.6
Fat Mass (kg)	13.1 ± 7.7
Fat Free Mass (kg)	69.1 ± 8.3
Muscle Mass (kg)	65.7 ± 8.0
BMI (kg/m^2^)	25.7 ± 4.3
1RM Back Squat (kg)	138.1 ± 30.8
Lifting Experience (years)	4.5 ± 2.4

BMI, body mass index; 1RM, one repetition maximum

### Experimental Procedures

#### Familiarization Session

During familiarization participants were provided with an overview of the study and informed of the risks of taking part in the study prior to providing their written informed consent. Following completion of a general health history questionnaire and the Physical Readiness Questionnaire (PAR-Q) participants’ anthropometric measurements including body height (cm), body mass (kg), fat free mass (kg) and fat mass (% and kg) were performed. Body height was measured using a physician’s scale (Detecto, Webb City, MO). Body mass and body composition (fat and lean mass) were measured using a Tanita bioelectrical impedance analyzer (BIA) (MC-980Uplus, Tanita Corporation of America, Arlington Heights, Illinois) following manufacturer procedures. The weight of the participants’ shorts and t-shirts was estimated at 0.5 kg and was entered into the BIA. Participants were instructed to remove their shoes and socks and then to stand on the BIA for approximately 30 s until the analysis was complete. Participants were then familiarized with the 4-7-8 SB technique which involved inhaling through the nose for 4 s, holding their breath for 7 s and then exhaling through the mouth for 8 s (Eskici İlgin and Yayla, 2023). Participants practiced several cycles of this breathing protocol while seated at rest with guidance from the research team until comfortable with the protocol.

Participants’ 1RM SQ was then determined following NSCA guidelines (Haff and Triplett, 2016). All participants performed a standardized dynamic warm-up prior to the 1RM protocol. After a warm-up of 5–10 repetitions with a standard 20-kg Olympic bar, participants completed 5–7 repetitions at approximately 75% of their estimated 1RM followed by 3–5 repetitions at approximately 85% of their 1RM and finally 1–2 repetitions at approximately 95% of their 1RM before 1RM attempts. A rest interval of 2–4 min was provided between each set and 1RM attempts continued with progressive loads until participants could not successfully complete the lift. After an unsuccessful attempt 7–9 kg was removed from the bar and following a 2–4-min rest interval another 1RM attempt was made. Following these procedures all 1RMs were established within 3–5 testing sets.

Following 1RM trials, participants performed two submaximal practice sets of 10 repetitions at 55% 1RM SQ with the SB protocol used between sets. The SB protocol was queued by the iBreathe app (Jade Lizard Software LLC) on an iPad (Apple, Inc., USA) which provided audio and visual cues for the timing of each phase of the breathing protocol for participants to follow along with. During these practice sets participants were instructed to perform repetitions at maximum concentric velocity as would be expected during the experimental sessions.

#### Experimental Sessions

The first experimental session was within 7 days of 1RM testing, but no sooner than 3 days (Hester et al., 2014). The sessions were identical starting with initial data collection including a compliance questionnaire to ensure adherence to experimental session instructions. Participants were then fitted with a Polar Heart Rate Monitor (Polar Electro, Kempele, Finland) using a chest strap and a resting HR was recorded after sitting quietly for 5 min. Resting blood pressure was then assessed manually (blood pressure cuff: Moore Medical LLC, Cardiology IV stethoscope: 3M Littman). A linear positioning transducer (TendoUnit, Tendo Sports Machines UK Ltd, London, UK) was attached to the right side of the barbell with the sensor unit directly under the barbell to ensure the string was perpendicular to the ground.

After completion of the standardized 5-min dynamic warm-up participants completed three additional warm-up sets of SQs, building from 50% to 70% of their 1RM with 3-min rest intervals between sets (Ribeiro et al., 2021). Following the warm-up, the load was set at 80% of 1 RM and the participant performed 5 sets of 3 repetitions with 3 min of rest between sets (Byrd et al., 2021; González-Badillo et al., 2005; Marques and González-Badillo, 2006). Participants were instructed to perform each repetition by lowering eccentrically for 2 s, taking a brief pause at the transition, and then completing the concentric phase as fast as possible. During the SB condition, participants breathed spontaneously for the first 1 min following each set and then performed the 4-7-8 method (4-s inhale, 7-s inhale hold, 8-s exhale) for the remainder of the recovery period (Eskici İlgin and Yayla, 2023).

During each set peak power, average power, and peak and average bar velocity were recorded for each repetition using the TendoUnit. The inter-set recovery HR (RHR) was calculated for each recovery period by subtracting the participant’s HR at the end of the 3-min recovery from their HR immediately post set. BP (systolic) was evaluated immediately post set and at the end of the recovery period with the difference between the two being calculated as the inter-set recovery BP (RBP). The RPE using the 10-point Omni Resistance training scale was recorded immediately after every set (Robertson et al., 2003). Additionally, after each 3-min recovery period participants subjectively rated their recovery status (RS) using the Perceived Recovery Status scale (Sikorski et al., 2013).

### Statistical Analysis

Statistical analysis was performed using SPSS version 28 (SPSS Inc., Chicago, IL). Statistical significance was set a prior at *p* ≤ 0.05. Descriptive statistics were calculated for each variable and data were tested for normality using the Shapiro-Wilk test. A two (condition: SB vs. CON) x five (sets: 1–5) repeated measures ANOVA was used to assess the effects of the breathing strategies on average power, average peak power, average velocity, average peak velocity, RBP, RHR, and RS. Bonferroni post-hoc analysis was used to further examine significant interaction and main effects. The assumption of sphericity was confirmed using the Mauchly's test. Greenhouse-Geisser epsilon corrections were used when the sphericity assumption was violated. Effect sizes were calculated using partial eta squared (η_p_^2^) (small = 0.01, medium = 0.06, large = 0.14).

## Results

### Average Power

**Figure 1:**
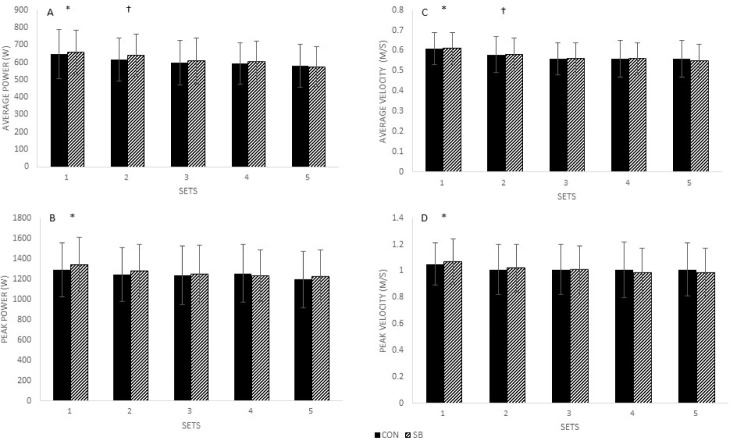
Average and peak power (W) (A&B respectively) and velocity (m/s) (C&D respectively) between sets of SQs for CON and SB. W, Watts; M/S, meters per second; SQ, barbell back squat; CON, control; SB, slow breathing. A: *, set 1 significantly greater than sets 2, 3, 4, and 5 (p ≤ 0.041); †, set 2 significantly greater than sets 3, 4 and 5 (p ≤ 0.006). B: *, set 1 significantly greater than set 3 (p = 0.038). C: *, set 1 significantly greater than sets 2, 3, 4, and 5 (p ≤ 0.021); †, set 2 significantly greater than sets 3, 4 and 5 (p ≤ 0.041). D: *, set 1 significantly greater than sets 2, 3, 4, and 5 (p ≤ 0.049).

There was no significant interaction between condition and sets (*p* = 0.226), nor was there a significant main effect for condition (*p* = 0.639) for average power. There was a significant main effect of sets (*p* < 0.001, n_p_^2^ = 0.63). Set 1 resulted in greater average power (653.1 ± 127.6 W) than sets 2 (627.2 ± 113.9 W), 3 (601.4 ± 117.9 W), 4 (598.0 ± 105.9 W) and 5 (576.6 ± 108.4 W) (all *p* ≤ 0.041). Set 2 average power was significantly greater than sets 3, 4 and 5 (all *p* ≤ 0.006). There were no differences in average power between sets 3, 4 and 5 (all *p* > 0.05) (Figure 1A).

### Peak Power

There was no significant interaction between condition and sets (*p* = 0.287), nor was there a significant main effect for condition (*p* = 0.779) for average peak power. There was a significant main effect of sets (*p* = 0.003, n_p_^2^ = 0.21). Only set 1 resulted in greater average peak power (1316.4 ± 250.6 W) than set 3 (1245.1 ± 259.0 W) (*p* = 0.038). There were no significant differences among sets 1, 2 (1261.8 ± 246.9 W), 4 (1242.1 ± 239.9 W), and 5 (1209.3 ± 260.7 W) (all *p* ≥ 0.078) (Figure 1B).

### Average Velocity

There was no significant interaction between condition and sets (*p* = 0.464), nor was there a significant main effect for condition (*p* = 0.773) for average velocity. There was a significant main effect of sets (*p* < 0.001, n_p_^2^ = 0.471). Set 1 resulted in significantly greater average velocity (0.61 ± 0.08m/s) than sets 2 (0.58 ± 0.08 m/s), 3 (0.56 ± 0.08 m/s), 4 (0.56 ± 0.08 m/s) and 5 (0.55 ± 0.08 m/s) (all *p* ≤ 0.021). Set 2 average velocity was significantly greater than sets 3, 4 and 5 (all *p* ≤ 0.041). There were no differences in average velocity among sets 3, 4 and 5 (all *p* > 0.05) (Figure 1C).

### Peak Velocity

There was no significant interaction between condition and sets (*p* = 0.435), nor was there a significant main effect for condition (*p* = 0.872) for average peak velocity. There was a significant main effect of sets (*p* < 0.001, n_p_^2^ = 0.279). Set 1 resulted in significantly greater peak velocity (1.06 ± 0.16 m/s) than sets 2 (1.02 ± 0.18 m/s), 3 (1.01 ± 0.18 m/s), 4 (1.00 ± 0.19 m/s) and 5 (1.00 ± 0.18 m/s) (all *p* ≤ 0.049). There were no differences in average peak velocity among sets 2, 3, 4 and 5 (all *p* > 0.05) (Figure 1D).

### Recovery Heart Rate

**Figure 2:**
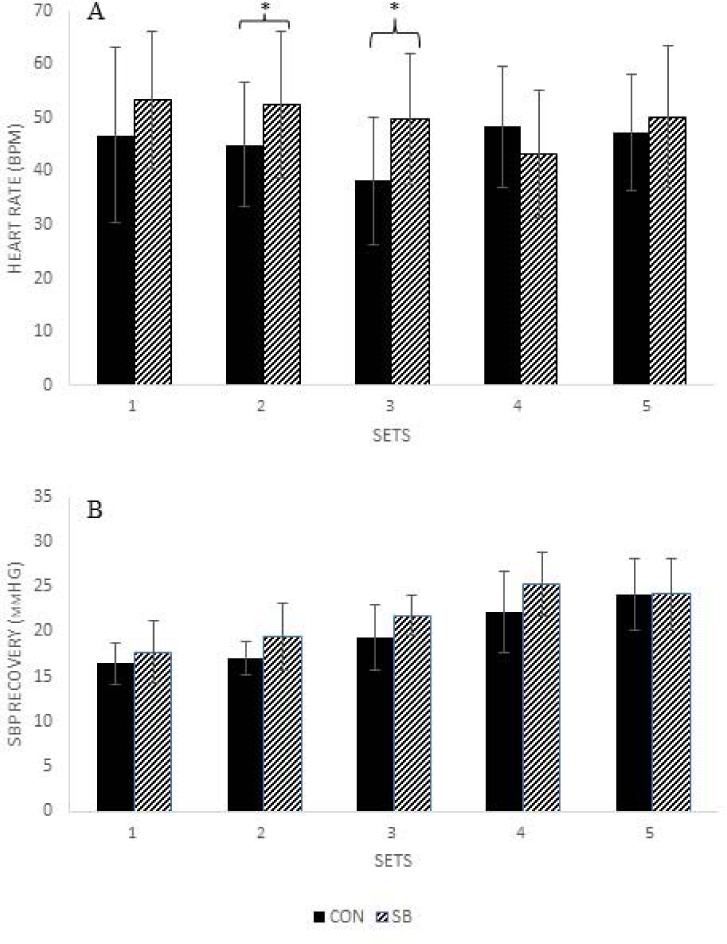
Recovery heart rate (BPM) (**A**) and systolic blood pressure recovery (mmHG) (**B**) between sets of SQs for CON and SB. BPM, beats per minute; SBP, systolic blood pressure; mmHG, millimeters of mercury; SQ, barbell back squat; CON, control; SB, slow breathing; *, SB significantly greater than CON (p ≤ 0.025).

There was a significant interaction between condition and sets (*p* < 0.001, n_p_^2^ = 0.413) on the recovery HR. There was a significant difference between conditions for set 2 (SB: 51.0 ± 14.9 bpm vs. CON: 44.5 ± 11.5 bpm, *p* = 0.025) and 3 (SB: 48.3 ± 13.5 bpm vs. CON: 37.7 ± 11.7 bpm, *p* = 0.006) with no differences in RHR between conditions for sets 1 (SB: 53.5 ± 12.8 bpm vs. CON: 46.8 ± 16.4 bpm, *p* = 0.056), 4 (SB: 41.7 ± 13.2 bpm vs. CON: 47.3 ± 12.0 bpm, *p* = 0.083) and 5 (SB: 49.9 ± 12.9 bpm vs. CON: 46.9 ± 10.6 bpm, *p* = 0.305). There was no significant main effect of condition (*p* = 0.089) on RHR. There was a significant main effect of sets (*p* = 0.009, n_p_^2^ = 0.219). RHR following set 1 (50.1 ± 13.0 bmp) and set 2 (47.8 ± 12.1 bmp) was significantly greater than following set 3 (43.0 ± 10.4 bpm) (*p* = 0.014 and 0.008, respectively). There were no differences in RHR between sets 4 and 5 (all *p* ≥ 0.127) (Figure 2A).

### Blood Pressure

There was no significant interaction between condition and sets (*p* = 0.964), nor was there a significant main effect for condition (*p* = 0.452) for RBP. There was a significant main effect of sets (*p* = 0.027, n_p_^2^ = 0.238), however, pairwise comparisons did not reveal any significant differences among sets (all *p* > 0.05) (Figure 2B).

### RPE

**Figure 3:**
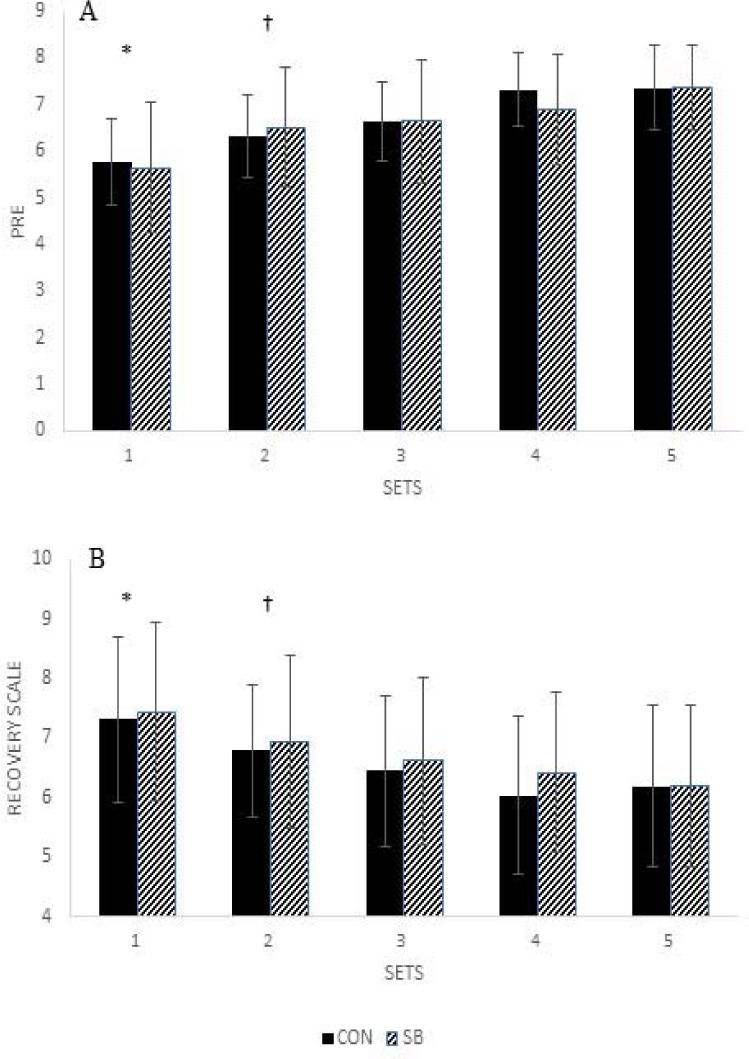
RPE (**A**) and the perceived recovery score (**B**) between sets of SQs for CON and SB. RPE, rating of perceived duration; SQ, barbell back squat; CON, control; SB, slow breathing. A: *, set 1 significantly higher than sets 2, 3, 4, and 5 (p ≤ 0.001); †, set 2 significantly higher than sets 3, 4, and 5 (p < 0.001). B: *, set 1 significantly greater than sets 2, 3, 4, and 5 (p ≤ 0.005); †, set 2 significantly greater than sets 3, 4 and 5 (p ≤ 0.025).

There was no significant interaction between condition and sets (*p* = 0.111), nor was there a significant main effect for condition (*p* = 0.653) for the RPE after each set. There was a significant main effect of sets (*p* < 0.001, n_p_^2^ = 0.671). Set 1 resulted in a significantly lower RPE (5.7 ± 1.1) than sets 2 (6.4 ± 0.9), 3 (6.6 ± 1.0), 4 (7.1 ± 0.8) and 5 (7.4 ± 0.9) (all *p* ≤ 0.001). The set 2 RPE was significantly lower than sets 4 and 5 (*p* < 0.001) with no difference between set 3 (*p* = 0.527). The set 3 RPE was significantly lower than sets 4 (*p* = 0.42) and 5 (*p* = 0.006). There were no differences in the RPE between sets 4 and 5 (all *p* > 0.05) (Figure 3A).

### Perceived Recovery Score

There was no significant interaction between condition and sets (*p* = 0.327), nor was there a significant main effect for condition (*p* = 0.211) for the RS after each set. There was a significant main effect of sets (*p* < 0.001, n_p_^2^ = 0.532). Set 1 resulted in a significantly higher RS (7.4 ± 1.4) than sets 2 (6.8 ± 1.2), 3 (6.5 ± 1.3), 4 (6.2 ± 1.3) and 5 (6.2 ± 1.4) (all *p* ≤ 0.005). The set 2 RPE was significantly higher than sets 3, 4 and 5 (all *p* ≤ 0.025). There were no differences in the RPE among sets 3, 4 and 5 (all *p* > 0.05) (Figure 3B).

## Discussion

We examined the use of a SB inter-set recovery strategy on power and bar velocity during sets of SQs in recreationally resistance trained males. Although SB was well tolerated by participants, there were no differences in peak or average power and bar velocity across sets compared to normal breathing. Additionally, there were no differences in perceptual measures of effort and recovery and SBP. However, SB improved HR recovery during sets 2 and 3 (of 5) to a greater degree than normal breathing. Lower recovery heart rates are often suggestive of enhanced recovery and worth further investigation with respect to SB strategies; however, whether lower inter-set recovery heart rates are advantageous or not is unclear, especially in multiple-set training strategies where optimal physiological recovery states have yet to be established.

Exercise has been shown to increase SNS activity and PSNS withdrawal followed by a reactivation of vagal tone at the cessation of exercise (Weberruss et al., 2018). The degree of withdrawal and the rate of recovery of PSNS activity depend on several factors including the intensity and duration of exercise (Michael et al., 2016, 2017; Weberruss et al., 2018). In contrast, SB reduces sympathetic tone and increases PSNS activity at rest (Magnon et al., 2021; Mason et al., 2013; Russo et al., 2017). SB following exercise has been shown to accelerate the parasympathetic recovery (Burg, 2020; Sugimoto et al., 2015). However, Burg (2020) notes that this enhanced recovery diminishes following the cessation of SB.

We hypothesized that SB using the 4-7-8 method would accelerate inter-set recovery and attenuate reductions in power and velocity performance set to set. The 4-7-8 method has gained popularity as a strategy to help improve sleep (Eskici İlgin and Yayla, 2023) in addition to the HR, BP and vagal tone (increased HRV). Although HR recovery was improved for the middle sets (sets 2 and 3) of SQs, this did not mitigate the normal set to set performance reductions or alter BP response compared to normal breathing. As this is the first study to our knowledge to investigate the SB strategy’s impact on resistance training outcomes, there may be many reasons for these findings. Michael et al. (2016, 2017) suggest that longer duration and higher intensity exercise result in greater reductions in PSNS activity. It is possible that the duration of exertion (3 repetitions) and/or intensity of effort (average RPE of 6.6 for SB and 6.7 for CON) in the present study was not sufficient to elicit a significant reduction in PSNS activity and therefore limited the effectiveness of SB.

Our results may have also been influenced by the duration of the SB protocol and modality of exercise. SB is a novel potential inter-set recovery strategy. It is possible that the duration of SB may have been too short or too long. In the present study a 3-min recovery period was used in which participants breathed spontaneously for the first 1 min and then performed the 4-7-8 method for the remaining 2 min. More research is needed to determine if this duration of SB was sufficient. Sakamoto et al. (2018) investigated the use of cyclic hyperventilation (HV) as an inter-set recovery strategy and found that 30 s of HV was superior to 15 s and 45 s of HV suggesting optimal duration for that breathwork strategy. The mode of exercise may also have impacted our results. Most studies investigating the ergogenic effects of inter-set breathwork or post-exercise recovery breathwork have used aerobic modalities (cycling, sprinting, etc.) (Burg, 2020; Sakamoto et al., 2013, 2015; Sugimoto et al., 2015). Only Sakamoto et al. (2022) and Buxton et al. (2022) have specifically investigated inter-set breathwork for resistance training outcomes. Although no performance or perceptual differences were noted for our study, the improvement in HR recovery suggests a potential for acute ergogenic effects as well as potential improvements in session-to-session recovery.

A primary limitation of the present study is the exclusion of any measures of PNS activity such as HRV. SB has been shown to increase HRV at rest and to accelerate reactivation of vagal tone following exercise (increased HRV compared to normal breathing). Future studies should therefore include continuous HRV measurement to assess the vagal response to resistance exercise and SB between sets. Additionally, research should investigate different methods of SB (cyclic sighing, box breathing, etc.) including the timing and duration of the SB interventions. Finally, in addition to identifying potential acute within-workout benefits of SB future investigations could assess inter-set SB strategies’ impact on recovery between workouts.

## Conclusions

Power is a critical element in sport and decreasing normal reductions in power and bar velocity during training through highly accessible strategies such as breathwork between sets would optimize training outcomes. We investigated the use of SB using the 4-7-8 method on peak and average power and bar velocity during 5 sets of 3 repetitions of SQs at 80% 1RM. SB was well tolerated by participants and improved HR recovery during the middle sets compared to normal breathing, but did not mitigate reductions in peak and average power and bar velocity from set to set. SB’s physiological effects suggest a potential to improve acute and longer-term recovery, however, more research is needed to identify specific training outcomes that benefit most as well as optimal timing and duration of SB.
